# An updated role of microRNA-124 in central nervous system disorders: a review

**DOI:** 10.3389/fncel.2015.00193

**Published:** 2015-05-20

**Authors:** Yang Sun, Zhu-Min Luo, Xiu-Ming Guo, Ding-Feng Su, Xia Liu

**Affiliations:** Department of Pharmacology, School of Pharmacy, Second Military Medical University, ShanghaiChina

**Keywords:** microRNA-124, CNS disorders, brain development, neurodegradation, CNS stress, neuroimmunity, brain tumor, stroke

## Abstract

MicroRNA-124 (miR-124) is the most abundant miRNA in the brain. Biogenesis of miR-124 displays specific temporal and spatial profiles in various cell and tissue types and affects a broad spectrum of biological functions in the central nervous system (CNS). Recently, the link between dysregulation of miR-124 and CNS disorders, such as neurodegeneration, CNS stress, neuroimmune disorders, stroke, and brain tumors, has become evident. Here, we provide an overview of the specific molecular function of miR-124 in the CNS and a revealing insight for the therapeutic potential of miR-124 in the treatment of human CNS diseases.

## Introduction

The discovery of microRNAs (miRNAs) introduced an understanding of the novel type of regulatory control over gene expression during plant and animal development (Ambros, 2004; Bartel, 2004). Mature miRNAs are non-coding transcripts of 18–25 nucleotides, which imperfectly bind to complementary sequences in the 3′-untranslated regions (UTRs) of target mRNAs to negatively regulate target gene expression. It is predicted that one third of human genes are miRNA targets (Lewis et al., 2005).

MicroRNA-124 (miR-124) is highly and specifically expressed in all brain regions except for the pituitary gland, and at 100 times lower expression in other tissues (Mishima et al., 2007; Baroukh and Van Obberghen, 2009). It was first identified in mice (Lagos-Quintana et al., 2002), and mature miR-124 is wholly homologous in mice, rats, and human. miR-124 has been reported to participate in chronic stress, neurodegeneration, alcohol/cocaine neuroadaptation, synapse morphology, neurotransmission long-term potentiation, neurodevelopment myeloid cell function, and hematopoiesis (Soreq and Wolf, 2011). In mammalian neurons, miR-124 suppresses the levels of 100s of non-neural genes, which contributes to the acquisition and maintenance of neuronal identity (Lim et al., 2005; Conaco et al., 2006). Furthermore, when miR-124 is aberrantly expressed, it contributes to pathological conditions involving the central nervous system (CNS) system. It has also been shown to be useful as a diagnostic and prognostic indicator of CNS disorders, such as brain tumor and stroke. Here we review the role and potential therapy of miR-124 in CNS development and disorders.

## miR-124 in CNS Development

Central nervous system development refers to the processes that generate, shape, and reshape the nervous system, from the earliest stages of embryogenesis to death. There has been particular attention on miRNAs in the context of mammalian nervous system development. Smirnova et al. (2005) compared miRNAs expression in embryonic neuron and astrocyte cultures, and found miR-124, a brain-enriched miRNA, preferentially expressed in neurons. miR-124 expression gradually increased and accumulated in parallel to neuronal maturation (Smirnova et al., 2005) during CNS development (Deo et al., 2006; Krichevsky et al., 2006). Blocking miR-124 activity in mature neurons leads selectively to increased levels of non-neuronal transcripts (Conaco et al., 2006), while increasing miR-124 activity in non-neuronal HeLa cells showed a shift of expression profile toward that of neuronal phenotype (Lim et al., 2005). Makeyev et al. (2007) demonstrated that miR-124 promoted this shift by triggering brain-specific alternative pre-mRNA splicing. In mouse embryonic development, miR-124 directly targets polypyrimidine-tract-binding protein (PTBP) mRNA, which encodes a global repressor of alternative pre-mRNA splicing in non-neuronal cells (Makeyev et al., 2007). During neuronal differentiation, miR-124 reduces PTBP levels, leading to the transition from non-nervous system to nervous system specific alternative splicing patterns. Interestingly, repression of PTBP is sufficient to induce trans-differentiation of fibroblasts into functional neurons (Xue et al., 2013).

Moreover, microRNA-124 is a subventricular zone (SVZ) neuronal fate determinant. The SVZ is the largest neurogenic niche in the adult mammalian brain. Knockdown of endogenous miR-124 maintained purified SVZ stem cells as dividing precursors, whereas ectopic expression led to increased neuron formation. Sox9 was demonstrated to be the physiological target of miR-124 responsible for this role (Cheng et al., 2009; Akerblom et al., 2012). Neurogenesis in embryonic neuroepithelial cells in the spinal cord is also regulated by miR-124 and its target Sox9 (Farrell et al., 2011). Several other targets of miR-124 have been identified in mediating the process of neurogenesis. For example, miR-124 in SVZ progenitor cells mediates stroke-induced neurogenesis by targeting the JAG-Notch signaling pathway in adult rats (Liu et al., 2011b). miR-124 also ensures the transition from neural progenitors to neurons by repressing two endogenous targets, LAMC1 and ITGB1, which are highly expressed by neural progenitors, but are repressed upon neuronal differentiation in the chick embryos (Cao et al., 2007). SCP1 down-regulation is another critical factor for inducing neurogenesis during embryonic CNS development in both chick and mouse embryos, and miR-124 contributes to this process in part by down-regulating SCP1 expression (Visvanathan et al., 2007).

Besides effects on neuron fate, miR-124 also contributes to the control of neurite outgrowth during neuronal differentiation possibly by cytoskeleton regulation in mouse P19 cells (Yu et al., 2008), and affects dendritic differentiation by regulating RhoG (Schumacher and Franke, 2013). Overexpression of miR-124 could lead to reduced astrocytic lineage differentiation by inhibiting STAT3 signaling (Krichevsky et al., 2006). miR-124 is essential for hippocampal axogenesis and retinal cone survival, as it represses Lhx2 translation in mice (Sanuki et al., 2011). In post-mitotic neurons, miR-124 represses BAF53a, which is essential for an evolutionarily conserved program of post-mitotic neural development and dendritic morphogenesis in mouse embryos (Yoo et al., 2009).

In general, miR-124 has been shown to promote cell differentiation and repress cell proliferation. In neuroblasts, miR-124 was highly expressed in cells at G_0_/G_1_ phase (Cheng et al., 2009), which results in the repression of Cdk6, a protein mediating cell-cycle progression from G_0_/G_1_ (Silber et al., 2008). miR-124 is expressed from the beginning of eye development in *Xenopus*, and has been shown to repress cell proliferation in the optic cup. However, this is not true during earlier development. It was recently reported that miR-124 is both necessary and sufficient to promote cell proliferation and repress neurogenesis at the optic vesicle stage which precedes optic cup formation, showing an anti-neural role by negatively regulating the expression of the pro-neural marker NeuroD1, and revealing a novel regulatory role of miR-124 in neural development (Liu et al., 2011a).

## miR-124 in Neurodegeneration

Memory storage and memory-related synaptic plasticity rely on precise spatiotemporal regulation of gene expression. miR-124 plays a critical role in the regulation of signaling molecules underlying synaptic plasticity and memory (Fischbach and Carew, 2009). miR-124 was exclusively present presynaptically in a sensory-motor synapse where it constrains serotonin-induced synaptic facilitation through regulation of the transcriptional factor CREB in *Aplysia*, suggesting a role for miR-124 in long-term plasticity of synapses in the mature nervous system (Rajasethupathy et al., 2009). miR-124 regulates cocaine-induced plasticity by targeting BDNF in rats, which is well implicated in synaptic plasticity and plays a central role in reward and memory (Chandrasekar and Dreyer, 2009). Thus, miR-124 may be involved in neurodegeneration diseases such as Alzheimer’s disease (AD) and Parkinson’s disease (PD).

Alzheimer’s disease is an age-related chronic neurodegenerative disease and the most prevalent type of dementia in elderly people. miR-124 level is down-regulated (human-originated, *n* = 5; Lukiw, 2007), while the expression of β-site APP cleaving enzyme 1 (BACE1) is up-regulated, in AD patients’ brain (Bigl et al., 2000; Sun et al., 2002; Yang et al., 2003; Lukiw, 2007; Smith et al., 2011), indicating a possible inverse relationship between them. Fang et al. (2012) further demonstrated that miR-124 overexpression or knockdown could decrease or increase the expression of BACE1, and found that miR-124 may work as an important regulating factor to alleviate cell death in the process of AD by targeting BACE1 in rat PC12 cells (Fang et al., 2012), which is considered to participate in the rate-limiting step in the production of neurotoxic Aβ (Hebert et al., 2008). Melatonin levels are decreased in the serum of AD patients, and its supplementation is able to reverse AD pathology and memory deficits. Wang et al. (2013) demonstrated that melatonin rescues the EPACs/miR-124/Egrl signaling pathway in rats, which is important in learning and memory (Yang et al., 2012).

Non-coding miRNAs are necessary for the survival of post-mitotic cells such as neurons that die in PD and other brain diseases. Kim et al. (2007) showed that mice lacking Dicer in specific dopamine neurons develop a progressive loss of neurons later in life, displaying a Parkinson’s-like disease. Thus, miRNAs are essential for maintaining dopaminergic neurons in the brain, and participate in the pathogenesis of PD. Furthermore, a recent review has shown that one–fourth (49 out of 202, MIRECORDS database) of “validated” targets of miR-124 are de-regulated in PD (Sonntag, 2010), indicating an important role for miR-124 in the regulation of this disease.

## miR-124 in CNS Stress

A number of miRNAs are shown to have important roles in the regulation of stress responses, and indeed miRNAs are shown to impart robustness to stress responses. The capacity of miRNAs to inhibit 100s of transcripts that are activated by stress makes them candidates for stabilizers of the homeostatic state of the transcriptome (Manakov et al., 2012).

Mental stress modifies both cholinergic neuro-transmission and alternative splicing in the brain. Meerson et al. (2010) reported that stress changes rat brain miRNA profiles detected by microarray, and some of these stress-regulated miRNAs, including miR-124, regulate alternative splicing. Also, miR-124 is up-regulated in the rat hippocampus after acute immobilization stress (Meerson et al., 2010). Another study examined the effect of maternal separation stress on miRNA expression and found the miR-124 level is elevated in the prefrontal cortex of stressed mice at P14 (Uchida et al., 2010). Paraventricular nucleus (PVN) neurons are affected by psychological stress through immune response activation. However, miR-124 was not affected by stress in mouse PVN (Mckennirey, 2011). *In vitro*, miR-124 from primary cultured neurons was significantly increased in response to all four neuronal challenge response sets (transfection, KCl, kainite, aging; Manakov et al., 2012).

Hundreds of transcripts endogenously expressed in neurons with target sites for miR-124 are coordinately upregulated in a variety of neuronal stresses. Overexpression of miR-124 indeed significantly inhibits expression of 100s of stress-induced transcripts detected by microarray (Manakov et al., 2012). Glucocorticoid and its receptor (glucocorticoid receptor, GR) exert profound effects on a variety of physiological processes, including adaptation to stress. Acute or chronic stress decreased GR mRNA in the PVN (Noguchi et al., 2010). Vreugdenhil et al. (2009) found that miR-124 decreased GR protein level and GR-mediated events. miR-124 expression also varies over time during the stress hyporesponsive period, a neonatal period when GC signaling is modulated (Vreugdenhil et al., 2009). By regulating GRs, miR-124 can affect a variety of systemic stress responses.

## miR-124 in Neuroimmunity

MicroRNAs have unique expression profiles in cells of the innate and adaptive immune systems and have pivotal roles in the regulation of both cell development and function (O’Connell et al., 2010). Soreq and Wolf (2011) designated miRNAs which notably affect both immune and neuronal functions as NeurimmiRs. NeurimmiRs may act as ‘negotiators’ between the nervous and immune systems (Soreq and Wolf, 2011). The cholinergic anti-inflammatory pathway is a link between the brain and the immune system of the host in response to an immune challenge. This pathway controls the inflammatory response through interaction with peripheral α7 subunit–containing nicotinic acetylcholine receptors (α7AChR) expressed on macrophages (Pavlov et al., 2003). Our group found miR-124 is necessary for the cholinergic anti-inflammatory action by inhibiting the production of pro-inflammatory cytokines (Sun et al., 2013b), and miR-124 may act as an NeurimmiRs.

The p38 mitogen-activated protein kinases (MAPKs) are central regulatory nodes coordinating acute stress and inflammatory responses. Lawson et al. (2013) reported that expression of the p38α protein is suppressed in the brain by two neuron-selective miRNAs, miR-124, and -128. miR-124 may influence neuroimmunity by affecting p38α -mediated signaling.

Microglial cells are macrophages that are resident in the brain and spinal cord and form the frontline defense of the innate immune system. Utilizing a mouse model of multiple sclerosis (MS) and experimental autoimmune encephalomyelitis (EAE), Ponomarev et al. (2011) showed that microglia miR-124 expression decreased by ~70% during the course of the disease, while overexpression of miR-124 could promote microglia quiescence and suppress EAE by deactivating macrophages via the C/EBP-α-PU.1 pathway (Zhang et al., 2004). Treatment of mice with miR-124 at the onset of EAE substantially ameliorated clinical symptoms and enhanced recovery in mice (Ponomarev et al., 2011).

The ability to prevent and attenuate EAE has implications for miR-124 in the treatment of neurodegenerative diseases such as MS and AD, where microglial cells are thought to play an integral role in the inflammatory process (Conrad and Dittel, 2011). Studies have already documented inflammation in AD, PD, amyotrophic lateral sclerosis (ALS), MS, and a growing number of other nervous system pathologies (Glass et al., 2010). However, a major question is whether pharmacological inhibition of inflammation pathways will be able to safely reverse or slow the course of disease, which needs further investigation.

## miR-124 in Stroke

Plasma miRNAs have been investigated as biomarkers for various diseases, including stroke. Recently, microarray analyses were done to characterize the miRNA expression profile in various stroke models (Jeyaseelan et al., 2008; Dharap et al., 2009; Liu et al., 2010; Yuan et al., 2010; Weng et al., 2011). The abundance of miR-124 in the CNS has accelerated efforts to determine if it can be used as an effective stroke treatment.

Jeyaseelan et al. (2008) first reported an elevated level of miR-124 in the brain samples from rats with middle cerebral artery occlusion (MCAO) followed by 24 h reperfusion. One of its predicted targets, the VSNL1 gene, which is a neuronal calcium sensor protein identified as a specific and promising plasma biomarker of stroke patients (Laterza et al., 2006), was decreased in parallel with the increased miR-124 under the same conditions (Jeyaseelan et al., 2008). Our previous study found miR-124 is significantly increased in ischemic penumbra as compared with that in the non-ischemic area in MCAO mice. Accordingly, brain tissues from stroke-prone spontaneously hypertensive rats (SHR-SP) showed higher miR-124 levels than in spontaneously hypertensive rats (SHRs; Sun et al., 2013a). Markedly increased plasma miR-124 was also observed at 24 h after stroke for both transient and permanent occlusions in rats (Laterza et al., 2009; Weng et al., 2011). Consistently, miR-124 was predicted to suppress acetylcholinesterase (AChE; Nadorp and Soreq, 2014), whose plasma level was reduced in patients post ischemic stroke (Ben Assayag et al., 2010), indicating a physiological relevance between miR-124 and AChE. The above suggests that plasma miR-124 released from the infracted brain may be a promising candidate biomarker for stroke identification.

Although there is no correlation between the infarct size and plasma miR-124 level in rats after MCAO introduction (Weng et al., 2011), efforts have been made to explore whether miR-124 treatment is effective in stroke. Doeppner et al. (2013) showed that exogenous miR-124 reduced brain injury and functional impairment, enhanced neurovascular remodeling, and increased angioneurogenesis 8 weeks post-stroke in mice with MCAO, which is possible via the pathway involving Usp14-dependent REST degradation. Similarly, our previous study showed that miR-124 overexpression decreased the infarct area of MCAO mice (Sun et al., 2013a). The anti-apoptosis proteins Bcl-2 and Bcl-xL, key regulators in attenuating stroke-induced apoptotic cell death (Martinou et al., 1994; Wiessner et al., 1999; Graham et al., 2000), are found to be the targets of miR-124 in this protective role for stroke. miR-124 was also found to inhibit stroke-induced neurogenesis by targeting the JAG1/Notch signaling pathway (Liu et al., 2011b).

However, conflicting miR-124 stroke therapy results have also been reported. For example, Zhu et al. (2014) showed that knockdown of cerebral miR-124 reduced cell death and infarct size, and improved neurological outcomes by negatively regulating Ku70. Additionally, Liu et al. (2013) found that although a miR-124 mimic did not affect the infarct volume at 24 h after ischemia, inhibition of miR-124 effectively reduced the ischemic injury due to iASPP expression up-regulation. Therefore, the use of miR-124 as an effective stroke treatment necessitates further research.

## miR-124 in Brain Tumor

Distinct patterns of miR-124 expression have been observed in many cancers including glioblastomas (Silber et al., 2008). miR-124 expression is significantly decreased in anaplastic astrocytoma and glioblastoma relative in human patients to non-neoplastic brain tissue (Fowler et al., 2011; Hua et al., 2012; Lang et al., 2012; Ho et al., 2013), and is expressed at different levels in glioblastoma compared with normal brain (Silber et al., 2008; Godlewski et al., 2010). Overexpression of miR-124 induced morphological changes and neural differentiation in mouse neural stem cells and oligodendroglioma, accompanied by reduced self-renewal and tumorigenicity (Silber et al., 2008). In addition, the ectopic expression of miR-124 in a glioblastoma cell line resulted in significant inhibition of migration and invasion, suggesting that miR-124 may be a novel inhibitor of glioblastoma invasion (Fowler et al., 2011).

Several targets of miR-124 mediating this process have been identified. It was reported that miR-124 controls self-renewal and tumorigenic competence of human glioblastoma cells by targeting SCP1 and PTPN12 phosphatases (Conti et al., 2012; Lee et al., 2013). Xia et al. (2012) demonstrated that the tumor suppressor activity of miR-124 could by partly due to its inhibitory effects on glioma stem – like traits and invasiveness through down-regulation of SNAI2 in human. Others reported that miR-124 inhibits glioma cells migration and invasion by down-regulation of ROCK1 (An et al., 2013), and induced glioma differentiation by suppressing Twist and SNAI2 (Xie et al., 2012).

A clinical investigation also showed a negative correlation between miR-124 expression and a hypoxic gene signature in glioblastoma patient samples. Increased miR-124 expression affects the ability of tumor cells to survive under O_2_ and/or nutrient deprivation, while miR-124 re-expression increases cell death *in vivo* and enhances the survival of mice bearing intracranial xenograft tumors. miR-124 exerts this phenotype in part by directly regulating TEAD1, MAPK14/p38α, and SERP1, which are factors involved in cell proliferation and survival under stress (Mucaj et al., 2014). Besides glioblastoma, neuroblastoma is an embryonic tumor derived from the autonomic nervous system neural-crest tissues, and is the most common extracranial solid tumor in children. Huang et al. (2011) showed that miR-124 plays a pivotal role in neuroblastoma by targeting aryl hydrocarbon receptor (AHR), which may promote neuroblastoma cell differentiation. These studies indicate that miR-124 is a potential therapeutic target in brain tumor treatment.

## Conclusion

We have discussed the importance of miR-124 in neuronal development and function of the brain. Changes of miR-124 levels can serve as biomarkers that indicate the functional status of a normal brain, as well as progression of CNS diseases. Interestingly, it seems inverse effect of miR-124 in tumorogenic events and neurodegenerative processes. For example, miR-124 alleviates cell death in the process of AD by targeting BACE1, while increases cell death in glioblastoma by regulating TEAD1, MAPK14/p38α, and SERP1 (Mucaj et al., 2014). This might be relevant to different targets involved depending on the context of diseases, cells and surroundings. Moreover, its regulation of numerous targets (summarized in **Table 1**) classifies it as a suitable treatment for the complexity of pathophysiological events launched by multifactorial CNS diseases, which require therapies that are able to trigger a large set of orchestrated genes. Therefore, we believe miR-124 and other miRNAs that fulfill this criterion will replace single gene therapies for the treatment of these kinds of diseases in the near future.

**Table 1:** Targets of microRNA-124 (miR-124) in central nervous system (CNS).

Target gene name	Abbreviation	CNS disorder	Function in CNS
SRY-box transcription factor	Sox9	Neurogenesis	Neurogenesis in embryonic neuroepithelial cells in the spinal cord is regulated by miR-124 and its target Sox9 (Farrell et al., 2011).
Small C-terminal domain phosphatase 1	SCP1	Neurogenesis glioblastoma	SCP1 down-regulation induces neurogenensis, and miR-124 contributes to this process in part by targeting it (Visvanathan et al., 2007). miR-124 controls self-renewal and tumorigenic competence of human glioblastoma cells by targeting SCP1 and PTPN12 phosphatases (Lee et al., 2013).
LIM homeobox protein 2	Lhx2	Neurogenesis	Lhx2 is required for hippocampal formation, and miR-124 is essential for hippocampal axogenesis and retinal cone survival by repressing Lhx2 translation (Sanuki et al., 2011).
Jagged-1	JAG1	Neurogenesis	miR-124 targets JAG1/Notch signaling pathway to inhibit neurogenesis (Liu et al., 2011b).
ephrin-B1	EfnB1	Neurogenesis	EfnB1 is a target of miR-124 in neurogenesis, while miR-124 is itself regulated by EfnB1 in neural progenitor cells (Arvanitis et al., 2010).
Distal-less homeobox 2	DLX2	Neurogenesis	DLX2 is a miR-124 target and regulates generation of interneurons in the embryo and promotes neurogenesis (Liu et al., 2011b).
Synaptogyrin 2	Syngr2	Neuronal differentiation	SYNGR2 is down-regulated by miR-124 and is a non-neural paralogue of the neural-specific gene synaptogyrin 1 (Lim et al., 2005).
BRG1/brm-associated factor 53a	Baf53a	Neuronal differentiation	In post-mitotic neurons, miR-124 represses BAF53a, which is essential for an evolutionarily conserved program of post-mitotic neural development and dendritic morphogenesis (Yoo et al., 2009).
BRG1/brm-associated factor 45a	Baf45a	Neuronal differentiation	miR-124 inhibits neural progenitor cell specific BAF45a to reduce proliferation and induce differentiation (Yoo et al., 2009).
Polypyrimidine-tract-binding protein 1	PTBP1	Neuronal differentiation	During neuronal differentiation, miR-124 reduces PTBP levels, leading to the transition from non-nervous system to nervous system -specific alternative splicing patterns (Makeyev et al., 2007).
Signal transducer and activator of transcription 3	STAT3	Neuronal differentiation	STAT3 selectively enhances differentiation of neural precursors along a glial lineage. Overexpression of miR-124 could lead to the reduced astrocytic lineage differentiation by inhibiting STAT3 signaling (Krichevsky et al., 2006).
Laminin γ1	LAMC1	Neuronal differentiation	miR-124 ensures the transition from neural progenitors to neurons by repressing LAMC1, which is highly expressed by neural progenitors, contributing to the formation of basal laminae (Cao et al., 2007).
Integrin β1	ITGB1	Neuronal differentiation	miR-124 ensures the transition from neural progenitors to neurons by repressing ITGB1, which is highly expressed by neural progenitors, maintaining the structural integrity of basal laminae (Cao et al., 2007).
Neurogenic differentiation 1	NeuroD1	Neuronal differentiation	miR-124 shows an anti-neural role by negatively regulating the expression of the pro-neural marker NeuroD1 at the optic vesicle stage (Liu et al., 2011a).
Ras homology growth-related	RhoG	Neuronal differentiation	miR-124 inhibits RhoG expression and promotes axonal and dendritic branching (Schumacher and Franke, 2013).
cAMP responsive element binding protein	CREB	Synaptic plasticity	miR-124 constrains serotonin-induced synaptic facilitation through regulation of the transcriptional factor CREB, which is an activator of transcription required for long-term facilitation (Rajasethupathy et al., 2009).
Brain derived neurotropic factor	BDNF	Synaptic plasticity	miR-124 regulates cocaine-induced plasticity by targeting BDNF, which is implicated in synaptic plasticity and plays a central role in reward and memory (Chandrasekar and Dreyer, 2009).
β-site APP cleaving enzyme 1	BACE1	Neurodegeneration	miR-124 may work as an important regulating factor to alleviate cell death in the process of AD by targeting BACE1, which is considered to participate the rate-limiting step in the production of neurotoxic Aβ (Fang et al., 2012).
Glucocorticoid receptor	GR	Neuronal stress	miR-124 is a down-regulator of GR activity with a possible relevance for brain GC signaling (Vreugdenhil et al., 2009).
CCAAT/enhancer-binding protein-α	C/EBP-α	Neuroimmunity	Overexpression of miR-124 could promote microglia quiescence and suppresses EAE by deactivating macrophages via the C/EBP-α-PU.1 pathway (Zhang et al., 2004; Ponomarev et al., 2011).
p38α mitogen-activated protein kinases (MAPKs)	p38αMAPK	Neuroimmunity	miR-124 may influence neuroimmunity by affecting p38α -mediated signaling, which are central regulatory nodes coordinating acute stress and inflammatory responses (Lawson et al., 2013).
Visinin-like 1	Vsnl1	Stroke	miR-124 level is elevated in the brain samples with MCAO followed by 24 h reperfusion, which is correlated with the repression of VSNL1 gene, a
			neuronal calcium sensor protein identified as a specific and promising biomarker in the plasma of stroke patients (Jeyaseelan et al., 2008).
Ubiquitin specific peptidase 14	Usp14	Stroke	miR-124 targets USP14 to suppresses deubiquitination of REST, which contributes to reduced brain injury and functional impairment, enhanced neurovascular remodeling, and increased angioneurogenesis 8 weeks post-stroke in mice with MCAO (Doeppner et al., 2013).
Inhibitory member of the apoptosis -stimulating proteins of p53 family	iASPP	Stroke	Inhibition of miR-124 effectively reduced the ischemic injury due to the up-regulation of iASPP level (Liu et al., 2013).
X-ray repair cross-complementing protein 6	Ku70	Stroke	Knockdown of cerebral miR-124 reduced cell death and infarct size, and improved neurological outcomes by negatively regulating Ku70, which is mainly involved in NHEJ of DSBs, V(D)J recombination, telomere maintenance, and regulation of Bax-mediated apoptosis (Zhu et al., 2014).
Protein tyrosine phosphatase, non-receptor type 12	PTPN12	Glioblastoma	miR-124 controls self-renewal and tumorigenic competence of human glioblastoma cells by targeting PTPN12 phosphatases (Conti et al., 2012; Lee et al., 2013).
TEA domain family member 1	TEAD1	Glioblastoma	miR-124 affects the ability of tumor cells to survive under O_2_ and/or nutrient deprivation in part by directly regulating TEAD1, which is among factors involved in cell proliferation and survival under stress (Mucaj et al., 2014).
Stress-associated endoplasmic reticulum protein 1	SERP1	Glioblastoma	miR-124 affects the ability of tumor cells to survive under O_2_ and/or nutrient deprivation in part by directly regulating SERP1, which is among factors involved in cell proliferation and survival under stress (Mucaj et al., 2014).
Neuroblastoma RAS viral (v-ras) oncogene homolog	NRAS	Glioblastoma	NRAS is among the oncogene targets of miR-124 in glioblastoma and its signaling pathway plays a crucial role in many cancers by regulating cell proliferation, differentiation, and survival (Lang et al., 2012).
Pim-3 proto-oncogene	PIM3	Glioblastoma	PIM3 is among the oncogene targets of miR-124 in glioblastoma and it promotes tumor cell growth through modulating cell cycle (Lang et al., 2012).
Rho-associated coiled-coil containing protein kinase 1	ROCK1	Glioma	miR-124 inhibits glioma cells migration and invasion by down-regulation of ROCK1, a well-known cell mobility-related gene (An et al., 2013).
Snail family zinc finger 2	SNAI2	Glioma	miR-124 suppresses SNAI2 to inhibit glioma invasiveness and induce glioma differentiation (Xia et al., 2012; Xie et al., 2012).
Twist family	Twist	Glioma	miR-124 induces glioma differentiation by suppressing Twist (Xie et al., 2012).
Aryl hydrocarbon receptor (AHR)	AHR	Neuroblastoma	miR-124 plays a pivotal role in neuroblastoma by targeting AHR, which may promote neuroblastoma cell differentiation (Huang et al., 2011).
Solute carrier family 16, member 1	Slc16a1	Medulloblastoma	SLC16A1 represents one of the non-neuronal targets regulated by miR-124, and its enhanced expression in medulloblastoma may confer growth advantage to tumor cells (Li et al., 2009).
Cyclin-dependent kinase 6	Cdk6	Medulloblastoma	Cdk6 mediates cell-cycle progression from G_0_/G_1_ (Silber et al., 2008).

## Conflict of Interest Statement

The authors declare that the research was conducted in the absence of any commercial or financial relationships that could be construed as a potential conflict of interest.
